# TLR Agonists as Modulators of the Innate Immune Response and Their Potential as Agents Against Infectious Disease

**DOI:** 10.3389/fimmu.2014.00079

**Published:** 2014-03-03

**Authors:** Edin J. Mifsud, Amabel C. L. Tan, David C. Jackson

**Affiliations:** ^1^Department of Microbiology and Immunology, The University of Melbourne at the Peter Doherty Institute for Infection and Immunity, Melbourne, VIC, Australia

**Keywords:** TLR agonists, immunomodulation, innate immunity, toll-like receptors, cytokines and inflammation

## Abstract

Immunotherapies that can either activate or suppress innate immune responses are being investigated as treatments against infectious diseases and the pathology they can cause. The objective of these therapies is to elicit *protective* immune responses thereby limiting the harm inflicted by the pathogen. The Toll-like receptor (TLR) signaling pathway plays critical roles in numerous host immune defenses and has been identified as an immunotherapeutic target against the consequences of infectious challenge. This review focuses on some of the recent advances being made in the development of TLR-ligands as potential prophylactic and/or therapeutic agents.

## Introduction

Second to clean water and improved hygiene practices, vaccination remains the most effective public health measure for the prevention and spread of infectious diseases. Vaccination is currently the most effective medical intervention for preventing infection and death from infectious diseases. The best evidence for this is the implementation of global vaccination regimes that have led to the eradication of smallpox and the vaccine-mediated control of a large number of other infectious diseases (1–3). Conventionally, vaccination induces long-lasting antigen-specific immunity and is employed *prophylactically* for the prevention of specific diseases. However, vaccines are not currently available against all human pathogens and even when available, the continuously evolving nature of some pathogens means that vaccination alone is not adequate for maintaining disease control. Immunotherapies that activate protective mechanisms of the innate immune system within the host are now also being investigated as potential prophylactic or therapeutic agents to combat infectious disease. With the development of anti-microbial resistance to antibiotics (4) and the need to update vaccines to accommodate pathogen evolution, the development of alternative forms of prophylactic agents with a broader spectrum of activity has emerged as a field of intense interest to the scientific community.

Promising new approaches to combat infectious disease involve modulation of the host’s innate immune system using ligands (agonists or antagonists), which bind to the receptor and perform agonist or antagonistic functions. Because these agents directly target the host rather than the pathogen, they are unlikely to result in the development of anti-microbial resistance even after repeated use. The rapid and broad nature of the innate immune system indicates that treatment with these agents will provide a broader spectrum of protection and could be used in combination with other anti-microbial agents including vaccines. The prophylactic administration of these agents could also be beneficial for those most susceptible to infections such as the elderly, who are poorly responsive to vaccination (5, 6).

Immunomodulating agents must induce a sufficient and appropriate immune response that limits inflammation and tissue injury and enables elimination of the pathogen. Additionally, the inflammatory response induced should not hinder the development of long-term antigen-specific immune responses, which will provide the host with continued protection in the event of subsequent exposure with the same or related pathogens. This aspect of long-term immunity is particularly important in the case of epidemics when re-infection rates are high.

## The Respiratory Mucosa: A Major Interface for Host–Pathogen Encounter

Innate immune responses at mucosal surfaces such as the respiratory and gastrointestinal tracts exhibit higher thresholds of activation, which prevent excessive inflammation to innocuous antigens (7). The respiratory system is an obvious and common target for both viral and bacterial infections and with the increasing incidence of drug resistance to commonly used antibiotics (4), immunomodulatory agents offer an alternative approach against respiratory infections.

In the airways of both man and mouse, there are numerous defense measures in place to combat invading respiratory pathogens. The lung mucosal layer contains specific humoral factors including collectins and defensins that act as a first line defense against infectious agents (8–10). Upon infection or stimulation with Toll-like receptor (TLR) ligands, lung respiratory epithelial cells increase production of anti-microbial peptides and are crucial in sensing and eliminating pathogens (11, 12). Respiratory epithelial cells along with other leukocytes express pattern recognition receptors (PRR) and sense pathogens displaying pathogen-associated molecular patterns (PAMP). These PAMPs can be expressed intra- as well as extracellularly (13). The most extensively characterized class of PRRs are the TLRs of which there are 10 members represented in the human genome (13–15). Other PRRs involved in viral and bacterial recognition include the nucleotide-binding oligomerization domain (NOD-like) receptor family, retinoic-acid-inducible gene-1 (RIG-1), and the inflammasome pathways.

**Figure 1:**
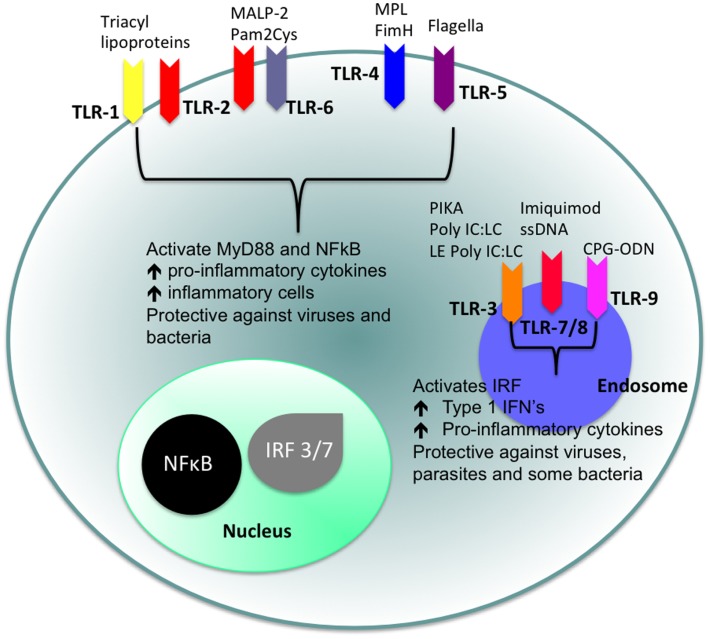
**Cellular location of TLRs and the identity of their ligands/agonists**. The stimulation of surface TLRs (TLR-2, TLR-4, and TLR-5) with appropriate ligands results in the activation of NFκB. The ensuing increase in levels of pro-inflammatory cytokines and the influx of inflammatory cells then provides an environment, which protects against both virus and bacterial challenge. Activation of intracellular TLRs (TLR-3, TLR-7, TLR-8, and TLR-9) leads to IRF activation and the production of Type 1 IFNs and pro-inflammatory cytokines, again providing an environment not conducive for pathogens.

Of particular interest to us in this review are the TLRs. TLRs are strategically placed on the cell surface or on the membrane of endocytic vesicles and other intra-cellular organelles and recognize a range of different PAMPs (15). The cellular localization of TLRs and the products of their activation are shown in Figure 1. TLR agonists include a range of naturally occurring ligands that include genetic material found in viruses, components of bacterial cell walls, and flagellin proteins. Surface TLRs such as TLR-1, TLR-2, TLR-4, TLR-5, TLR-6, and TLR-11 mainly recognize microbial membrane components including lipids, lipoproteins, and flagella. TLR-3, TLR-7, TLR-8, and TLR-9 are expressed in intra-cellular vesicles of the endoplasmic reticulum, endosomes, and lysosomes and recognize both microbial and viral nucleic acids.

**Figure 2:**
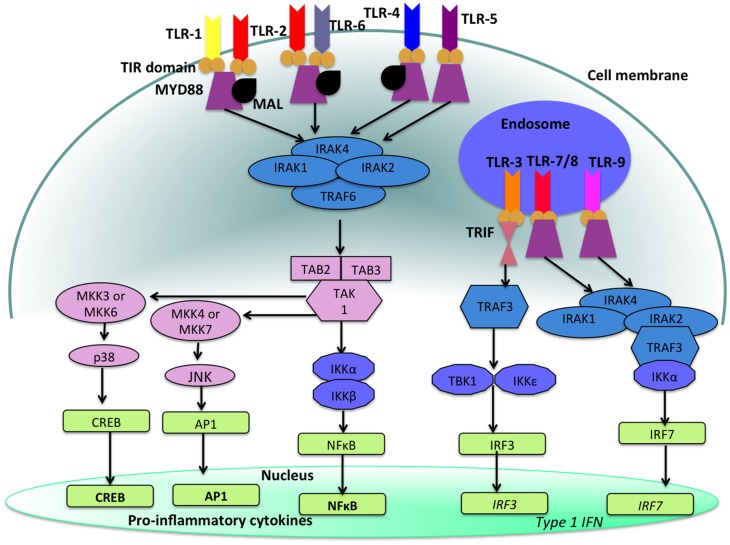
**TLR-signaling pathways**. TLR-4, TLR-5, and the heterodimers TLR-1/TLR-2 and TLR-2/TLR-6 are located on the cell surface where they are activated by the appropriate ligand. Conversely, TLR-3, TLR-7, TLR-8, and TLR-9 are located within endosomal compartments of the cell and recognize microbial and viral nucleic acids. Stimulation of TLR-1/TLR-2, TLR-2/TLR-6, TLR-4, and TLR-5 leads to the engagement of myeloid differentiation primary response protein (MyD88) and MYD88-adapter-like protein (MAL) with the TIR domain-containing adapter proteins. This stimulates downstream signaling pathways that involve the interactions between IL-1R-associated kinases (IRAKs) and the adapter molecules TNF receptor-associated factors (TRAFs) and activates mitogen-activated protein kinases (MAPKs) JUN N-terminal kinases (JNK) and p38. Activation of these kinases leads to the activation of transcriptional factors such as nuclear factor-κB (NF-κB), cyclic AMP-responsive element binding protein (CREB), and activator protein-1 (AP1). A major consequence of activation of surface TLRs is the induction of pro-inflammatory cytokines. Activation of TLR-7, TLR-8, and TLR-9 also leads to the engagement of MyD88, MAL, IRAKs, and IKKα, however, interferon-regulatory factors (IRFs) are activated, which leads to the production of type 1 interferons (IFN). Stimulation of TLR-3 results in the association of TIR domain-containing adapter protein inducing IFNβ (TRIF). This leads to the down stream signaling of TNF receptor-associated factors (TRAFs) and IKK leading to the activation of IRF3 and the production of type 1 IFNs. This image was adapted from Ref. (17).

With the exception of TLR-3, stimulation of a TLR by its ligand results in the activation of a signal transduction cascade that leads to the production of cytokines via the activation of the adapter molecule myeloid differentiation primary response differentiation gene 88 (MyD88) and nuclear factor-κB (NF-κB) (14, 16). TLR-3 utilizes MyD88-independent signaling pathways that employ the adapter molecule Toll/IL-1R (TIR) domain-containing adapter producing interferon-β (IFN-β) (TRIF). Activation of TLR-3 leads to the production of both Type 1 IFN as well as pro-inflammatory cytokines. Refer to Figure 2 for a more detailed description of TLR-signaling pathways.

## Targeting Cells of the Innate Immune System

The pulmonary tract is populated by a miscellany of innate immune cells, which are poised to dispose of pathogenic material and to initiate a variety of immune responses. These various cells are responsible for initiating inflammatory responses but they also work in concert to keep the innate immune system and its responses in check. For example, airway macrophages (AMΦ) exhibit an inhibitory phenotype (18), which limits unwanted and excessive inflammatory responses (19). By keeping the activation of the innate immune system in check, the lung can maintain homeostasis in an environment where there is continual exposure to antigens and other irritants.

During respiratory viral or bacterial infections, activation of TLRs on the surface of AMΦ results in cell activation (20), a mechanism that causes greater phagocytic activity of the cell, greater oxidative burst capabilities, and the secretion of pro-inflammatory instead of immunosuppressive cytokines (20). The secretion of cytokines and chemokines by AMΦ’s subsequently results in the recruitment and activation of other inflammatory cells into the lung. AMΦ possess this duality of effector functions in order to protect the respiratory tract from pathogenic agents and maintain integrity of the lung.

Neutrophils are the first cells of the innate immune system to respond to both viral and bacterial infections limiting pathogenic spread through secretion of cytokines and by antibody-dependent cell-mediated cytotoxic activity (ADCC) (21). Activation of natural killer cells (e.g., by macrophage-derived cytokines) induces secretion of IFN-γ, which stimulates macrophages to phagocytose material (22) and increases antigen presentation by antigen-presenting cells (APC) (23, 24). A key subset of APCs are the dendritic cells (DCs), which are said to bridge the gap between innate and adaptive immunity and following activation and maturation, DCs migrate to the lymph nodes where they encounter and prime naïve T-cells (25).

The ability to activate anti-bacterial/anti-viral activity through the TLR pathways means that TLR-ligands could be utilized as a novel approach against infectious disease (26–30). A disadvantage of stimulating an innate immune response through activation of TLRs is that inappropriate inflammatory responses can result causing tissue damage and even autoimmunity. Therefore, activating the immune system must be tightly regulated and induce *appropriate* inflammatory responses. It is not surprising that the development of TLR agonists as anti-microbial agents and adjuvants is now being accompanied by the development of TLR antagonists as reagents and drugs to dampen immune responses [for review, see (31)]. In this review, we focus on the ways in which TLR agonists have been harnessed as anti-microbial agents and as adjuvants by making use of their intrinsic ability to initiate an (inflammatory) innate immune response.

## Situations Where TLRs Play a Role in the Outcome of Infection

The significance of TLR-signaling in anti-microbial defense has been illustrated in various experimental models of infection [for recent reviews, see (32, 33)]. A list of TLR-ligands discussed in this review are described in Table 1. The role of MyD88 and TLR-signaling during bacterial and viral infections has been demonstrated in models of *Legionella pneumophila, Staphylococcus aureus*, and respiratory syncytial virus (RSV) infections (34–37). These studies have highlighted the importance of TLR-signaling in initiating MyD88-dependent responses that contribute to host protection. As well as the role played by individual receptors, different TLRs also work in concert to provide effective microbial control. For example Bafica et al. (38) have demonstrated that animals lacking TLR-9 and TLR-2 exhibited markedly enhanced susceptibility to *Mycobacterium tuberculosis* infection than animals lacking one or the other individual TLRs.

**Table 1:** **TLR targeting immunostimulatory agents being investigated in animal models**.

Immunomodulatory agent	TLR-target	Infectious target	Reference
Pam2Cys	TLR-2	Influenza A virus	Tan et al. (30)
MALP-2	TLR-2	*S. pneumoniae*	Reppe et al. (26)
Poly IC	TLR-3	*L. major* HSV-2	Zimmermann et al. (90), Harandi et al. (93)
Poly IC:LC and LE Ply IC:LC	TLR-3	Influenza A virus	Stephen et al. (54), Kende (55), Wong et al. (112), Wong et al. (113)
		Yellow fever virus	
		Rift valley fever	
		Rabies	
PIKA	TLR-3	Influenza A virus	Lau et al. (52)
MPL	TLR-4	*L. monocytogenes*	Persing et al. (67), Cluff et al. (69), Cole et al. (70)
		Influenza A virus	
		*F. tularensis*	
FimH	TLR-4	Influenza A virus	Abdul-Careem et al. (72)
AGP	TLR-4	*F. novicida*	Lembo et al. (75)
Flagellin protein	TLR-5	*S. pneumoniae*	Munoz et al. (78)
Imiquimod	TLR-7	HSV-2	
CpG-ODN	TLR-9	Influenza A virus	Krieg et al. (114), Zimmermann et al. (90), Elkins et al. (91), McCluskie et al. (96), Jiang et al. (115)
		*L. major*	
		*F. tularensis*	
		*HSV-2*	
Pam2CSK4 and ODN2395	TLR-2 and TLR-9	*S. pneumoniae, P. aeruginosa, B. anthrax, S. aureus*, influenza A virus	Clement et al. (98), Tuvim et al. (27), Evans et al. (99), Duggan et al. (100)

## TLR-2

TLR-2 forms heterodimers with both TLR-1 and TLR-6 and broadening the repertoire of ligands recognized by this receptor. For example, triacyl-lipoproteins activate TLR-2 in combination with TLR-1, whereas diacyl-lipoproteins are recognized by TLR-2 in combination with TLR-6 (39).

A well-characterized ligand and agonist for TLR-2 is macrophage-activating lipopeptide-2 (MALP-2), which was originally isolated from *Mycoplasma fermentans* (40). MALP-2 promotes the transient elevation of monocyte chemoattractant protein-1 (MCP-1), interleukin-8 (IL-8), macrophage inflammatory protein-1α (MIP-1α), MIP-1β, IL-6, and tumor necrosis factor-α (TNF-α) *in vivo* and *in vitro* (26, 41, 42). Intra-tracheal administration of MALP-2 in mice and rats also leads to activation and recruitment of neutrophils and macrophages into the lungs (26, 42). Furthermore, the administration of MALP-2, 24 h prior to challenge with *Streptococcus pneumoniae*, reduces pulmonary bacterial burden, which correlates with elevated levels of CCL5 and leukocyte migration (26).

**Figure 3:**
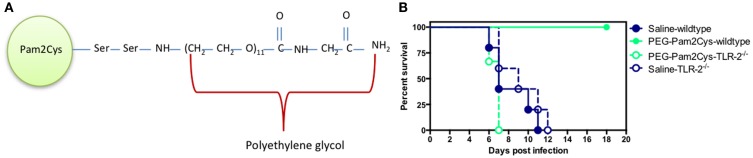
**Schematic diagram of (A) PEG-Pam2Cys and its effects (B) when administered intra-nasally to mice challenged with influenza virus**. Groups of five C57BL/6 (wild-type) and TLR-2^−/−^ knockout mice received 20 nmol of PEG-Pam2Cys or saline 3 days prior to challenge with 200 PFU of H1N1, PR8 influenza virus. Mice were monitored daily for survival.

A synthetic analog of MALP-2, S-[2,3-bis(palmitoyl oxy)propyl] cysteine (Pam2Cys) is a potent adjuvant that has been incorporated into a number of lipopeptide vaccine candidates (43–49). The addition of Pam2Cys to peptide-based vaccines is critical for their immunogenicity and enabling lipopeptides to both target and activate DCs (43, 50). More recently, a soluble version of Pegylated-Pam2Cys (PEG-Pam2Cys, see Figure 3A) was assembled in order to investigate the effects that Pam2Cys has on immunity when administered in the absence of antigen (30). In these studies, it was found that intra-nasal administration of PEG-Pam2Cys resulted in the influx of innate immune cells including neutrophils, macrophages/monocytes, NK-cells and γδ T-cells, and pro-inflammatory cytokines including IL-10, IL-6, TNF-α, IFN-γ, MCP-1, and IL-12p70 into the lungs of mice (30). This response reached a maximum of 3 days after treatment and returned to basal levels 7 days after administration. The significance of these pulmonary changes were that mice treated with PEG-Pam2Cys were protected against virulent H1N1 A/Puerto Rico/8/34 (PR8, Mount Sinai) influenza virus infection for up to 7 days following treatment (30) (Figure 3B). The effects mediated by Pam2Cys were dependent on TLR-2 because the effect was not apparent in TLR-2^−/−^ mice. Furthermore, the effects of Pam2Cys were dependent on intra-nasal administration under light anesthesia, which results in the delivery of Pam2Cys to the lungs (30). PEG-Pam2Cys treatment also reduced viral transmission rates following influenza infection suggesting that this treatment modality could reduce the spread of infection (30). We also demonstrated that treatment with Pam2Cys promotes the development of influenza-specific adaptive immune responses that included IFN-γ, TNF-α, and IL-2 secreting CD8^+^ T-cells, which were found to persist in the lung up to 6 weeks after infection (30). These results demonstrate that stimulation of the innate immune system with Pam2Cys not only provides immediate protection against challenge with influenza virus but also permits the development of influenza-specific immune responses that provide the host with continued protection following challenge.

## TLR-3

An agonist for TLR-3 is double-stranded RNA (51), which is associated with viral infection and engagement of the production of Type 1 IFNs (51) that have potent anti-viral actions.

A synthetic TLR-3 agonist, PIKA is a chemically stabilized analog of polyinosinic:polycytidylic acid (Poly IC), which itself is a structural analog of double-stranded RNA. Following three intra-nasal doses of PIKA, interstitial macrophages, neutrophils, and plasmacytoid DCs are recruited into the lungs and the levels of TNF-α, IFN-γ, keratinocyte chemoattractant (KC), and IFN-β are also elevated (52). The modified pulmonary environment induced by PIKA treatment significantly reduces viral burden in the lungs of mice infected with a variety of influenza viruses including A/teal/HK/W312/97 (H6N1), A/rhea/NC/93 (H7N1), A/HK/1073/99 (H9N2), and A/Vietnam/1203/2004 (H5N1), and an isolate from the 2009 H1N1 influenza pandemic, A/California/07/2009 (H1N1) (52). Additionally, PIKA is a potent adjuvant that has been shown to enhance both cellular and humoral immune responses to the surface protein of Hepatitis B (53).

Polyinosinic–polycytidylic acid (Poly IC) condensed with poly-l-lysine and carboxymethylcellulose (Poly IC:LC) is another chemically stabilized version of synthetic double-stranded RNA, which has been shown to activate TLR-3, resulting in the protection of mice and rhesus monkeys from infection with yellow fever, Rift Valley fever, and rabies viruses (54, 55). Intra-nasal administration of Poly IC:LC also provides a high level of protection against lethal challenge with the highly virulent avian influenza virus A/H5N1/chicken/Henan, clade 2, the lethal PR8 strain of influenza virus, and the A/Aichi/2 (H3N2) influenza virus (56, 57). Liposome encapsulation of Poly IC:LC (LE poly IC:LC) reduces the toxicity associated with Poly IC:LC in addition to prolonging the activity, of Poly IC, extending protection against lethal viral challenge from 14 days up to 21 days post treatment (58).

The anti-viral activity of TLR-3 agonists such as Poly IC:LC and LE Poly IC:LC rely on the ability of these ligands to stimulate the production of IFN-γ, α, and β. Although these cytokines are important during viral infections, their role during bacterial infections may not be as beneficial to the host. During the course of infection with *Mycobacterium tuberculosis*, it has been observed that Poly IC:LC treatment twice daily after infection enhances bacterial replication and induces extensive areas of necrosis in the lungs of treated mice compared to untreated mice (59). The exacerbation of disease was due to the secretion of type 1 IFN and the recruitment of CD11b^+^GR1^int^F4/80^+^MHCII^lo^ cells (59). Due to the promising effects observed with Poly IC:LC treatment during viral infections, new clinical applications of these drugs are likely to emerge but the data generated by the study of Antonelli et al. (59) urges caution in the use of immunomodulatory agents that stimulate Type 1 IFN in regions where tuberculosis is endemic.

Intra-nasal administration of two doses of Poly IC to mice has also been reported to increase the bacterial burden following infection with *S. pneumoniae* and methicillin-resistant *S. aureus* (60). Bacterial clearance is impaired by Type 1 IFN signaling which itself is dependent on the duration of Poly IC exposure (60). This study also suggests that in contrast to their anti-viral activity, Type 1 IFNs do not promote bacterial clearance. Following infection with influenza virus, elevated levels of Type 1 IFNs have been shown to leave mice more susceptible to infection with *S. pneumoniae* (61). The presence of Type 1 IFNs in the lung elevates bacterial burden through the attenuation of MIP-2 and KC production and neutrophil recruitment all of which facilitate bacterial elimination (61). Furthermore, the elevated levels of IFN-γ that follow influenza infection have been shown to inhibit AMΦ by effectively eliminating *S. pneumoniae* from the lungs (62). These viral–bacterial interactions are relevant to human infection as viral infections have been found to predispose the host to secondary bacterial infections (63), particularly during influenza A infections where *S. pneumoniae* has been identified as the most common organism causing secondary bacterial infections (64, 65).

## TLR-4

Lipopolysaccharide (LPS) is a bacterial membrane component specific to Gram-negative bacteria that activates TLR-4 (66). LPS is a potent immunomodulatory agent but due to its extreme toxicity, which includes the induction of sepsis-like symptoms, its clinical use is limited. Monophosphoryl lipid A (MPL) isolated from *Salmonella minnesota R595* induces similar cytokine profiles to LPS with less toxicity (67) furthering its investigations in clinical settings.

Vaccines formulated with MPL have been evaluated for their ability to enhance both systemic and mucosal immune responses against hepatitis B surface antigens, tetanus toxoid, and influenza (68). Vaccine formulations containing MPL induce both mucosal and systemic immune responses characteristic of a Th1-type response (68). The administration of MPL 2 days prior to or on the day of lethal influenza virus challenge was shown to reduce the mortality associated with infection (67). Cluff and colleagues went on to demonstrate that the intra-venous or intra-nasal inoculation of MPL 2 days prior to infection with either *Listeria monocytogenes* or lethal influenza virus provided protection in a TLR-4-dependent manner (69). MPL and MPL-mimetics could play a useful therapeutic role in modulating the innate immune response to provide short-term resistance to infectious challenge. Furthermore, the co-administration of *Francisella tularensis*-LPS and MPL or MPL alone were shown to protect mice from infection with *F. tularensis* (70). Protection against the intra-cellular pathogen was attributed to the differentiation of MPL-stimulated macrophages into “classically activated” macrophages (70), which have enhanced phagocytic activity and do not promote the survival of intra-cellular bacteria (71).

Fimbriae H protein (FimH) is a component of type 1 fimbriae and produced by uropathogenic *Escherichia coli* and is a ligand for TLR-4 (72). The administration of FimH to mice leads to the influx of neutrophils, TNF-α, RANTES, and IL-12 into the bronchoalveolar lavage (BAL) fluid of mice inducing pulmonary changes that reduces mortality and morbidity rates associated with influenza infection in a manner that is not critically dependent on AMΦ (72).

Aminoalkyl glucosaminide phosphates (AGPs) are synthetic mimetics of lipid A that require TLR-4 for recognition (73, 74). Intra-nasal administration of AGPs to mice induces the production of MIP-2, TNF-α, and IFN-γ (75). Mice prophylactically treated with AGPs prior to the inhalation of *F. novicida* displayed reduced bacterial burden in the lung, liver, and spleen following challenge and exhibited lower mortality rates in comparison to PBS-treated mice, which succumbed to infection 4 days later (75). Protection was dependent on IFN-γ, which amplified the innate immune response following treatment with AGPs. Furthermore, surviving mice developed both humoral and cellular adaptive immune responses, which fully protected mice from secondary challenge with the same microorganism (75).

## TLR-5

An agonist for TLR-5 is bacterial flagella proteins (76) and leads to the production of pro-inflammatory cytokines. *S. pneumoniae* infections are a major cause of pneumonia world-wide and the bacterium contains a range of PAMPs that activate TLR-2, TLR-4, TLR-9, but not TLR-5 (77). Flagellin isolated from *S. enterica* serovar typhimurium is not naturally associated with *S. pneumoniae* infections, but is a ligand for TLR-5 and promotes the survival of mice following lethal challenge with the bacterium (78). Treatment with flagellin recruits neutrophils as well as IL-6, TNF-α, CXCL-1, CXCL-2, and CCL20 into the airways. The protection afforded by this ligand is dependent on TLR-5 but not dependent on B-cells or T-cells (78).

As is the case with Pam2Cys, another TLR agonist which is associated with bacteria and which protects against viral infection, the results obtained with flagellin also highlight the fact that activation of TLRs can be used as therapeutic or prophylactic strategies to combat infectious diseases.

## TLR-7

TLR-7 recognizes single-stranded RNA fragments located in endosomes (79) and TLR-7 activation is a common feature of viral infections. 1-(2-methylpropyl)-1H-imidazo(4,5-c) quinoline-4-amine (Imiquimod) is a potent TLR-7 agonist, which enhances both the innate and adaptive immune pathways via the endogenous production of pro-inflammatory cytokines (80). Imiquimod was first approved by the Food and Drug Administration (FDA) in 1997 for the topical treatment of genital warts. Using the guinea pig model of herpes simplex virus-2 (HSV-2) infection, it was found that the intra-vaginal application of Imiquimod reduced the frequency of lesion development (81).

Infectious agents including viruses and some bacteria can cause cancer as reviewed in Ref. (82) and Mokhtari et al. (83) have implicated human papilloma viruses as possible etiologic agents in basal cell carcinomas. In this context, it is worth noting that the topical application of a cream containing 5% Imiquimod either once or twice daily for 3 days resulted in significantly enhanced clearance rates (84). The data suggest that immunostimulating agents can be used to treat cancer.

Topical Imiquimod is also extensively used to treat dermatologic conditions, however, patients can experience a range of side effects that in extreme cases can result in superficial scarring (85, 86) somewhat limiting its use. Therefore, variants of Imiquimod with reduced side effects could improve the utility of these compounds.

## TLR-9

CpG activates the innate immune system via TLR-9 and synthetic oligonucleotides displaying non-methylated CpG (CpG-ODN) motifs mimic the immunostimulatory effects of bacterial DNA (87–89). In Balb/c mice, infection with *Leishmania major* is a Th2-driven disease, which kills mice 8 weeks after challenge (90). The administration of CpG-ODN skews the immune response toward a Th1 immune response and mice treated with CpG-ODN are found to be resistant to infection with *L. major* (90). Elkins and colleagues (91) extended the finding of Zimmerman and showed that DNA motifs in the form of bacterial genomic DNA or synthetic ODN DNA confer protection against *L. major* and *F. tularensis* both of which are intra-cellular pathogens. Protection was independent of the route of inoculation and persisted for 2 weeks after treatment (91, 92), demonstrating the potential therapeutic application of this agent.

The importance of TLR-9 in conferring protection against HSV-2 infections has also been demonstrated by Harandi and colleagues (93) who showed that administration of CpG-ODN is effective against HSV-2 challenge. Treatment limits viral replication and increases survival following HSV challenge (94). CpG does not directly inhibit the virus but acts to stimulate immune responses (95). The efficacy of CpG-ODN over other TLR-ligands including TLR-3, 4, and 5 was dependent on these ligands’ ability to induce IFN-β, but not TNF-α, IFN-γ, or IFN-α (95), which suggests that protection is dependent on the TLRs ability to stimulate IFN-β.

The topical application of CpG-ODN and Resiquimod (R-848, a TLR-7/8 agonist) have been compared in a study for the prophylactic or therapeutic treatment of HSV-2 infection (96). Although prophylactic or therapeutic treatment with R-848 failed to provide any survival advantage (96), treatment with CpG-ODN either prophylactically or therapeutically protected mice from lethal HSV-2 challenge. Protection in CpG-ODN treated mice was attributed to the local cytokine response elicited by the TLR-9 ligand, whereas, R-848 induced a greater systemic chemokine response which was ineffective at controlling the virus. This demonstrates the importance of targeting the activity of TLR agonists to the site of infection whilst also limiting the extent of (systemic) inflammation in the circulation.

Although single administrations of CpG-ODNs are effective when used prophylactically against a range of bacterial and viral infections, for therapeutic use these compounds may need to be delivered multiple times. Daily administration of CpG-ODNs to mice causes considerable pathology in lymphoid organs and alterations in the structure and function of the lymphoid follicle (97). Additionally, after 3 weeks multi-focal liver necrosis and hemorrhagic ascites developed (97). Determining the balance between host defense functions and the potentially harmful effects of prolonged TLR activation will be crucial in the development of immunotherapies based on CpG, and presumably other, TLR-ligands.

## TLR-2 and TLR-9

We have so far discussed the use of single TLR-ligands in promoting protection against invading pathogens but the administration of *multiple* ligands has been shown to synergistically provide protection against respiratory pathogens. Non-typeable *Haemophilus influenza* (NTHi) lysate has been shown to provide broad spectrum protection against *Pseudomonas aeruginosa, Klebsiella pneumoniae, S. aureus, Bacillus anthrax, Yersinia pestis, S. pneumoniae*, and influenza virus (27, 98, 99). Protection conferred by NTHi was not dependent on leukocyte recruitment but depended on the stimulation of respiratory epithelial cells in the lung lining to secrete anti-microbial polypeptides (99).

Because the lysate contains a range of PAMPs, it was suggested that protection was at least partially dependent on TLRs. The effects mediated by NTHi are dependent on the adapter protein MyD88 and not TRIF (100). A range of TLR-ligands were tested to determine whether an individual ligand could confer protection similar to whole NTHi lysate. Although the administration of single TLR-ligand failed to induce resistance against *S. pneumoniae* challenge, a combination of TLR-2 and TLR-9 ligands (Pam2CSK4 and ODN2395, respectively), synergistically conferred protection against *S. pneumoniae* (100). The synergistic combination of these two TLR-ligands therefore provided a survival advantage against bacterial infection. In addition, administration of the synergistic multiple TLR-ligands is not only an effective anti-microbial strategy but also a new vaccine strategy being employed to enhance mucosal T-cell immune responses (101).

## Potential Side Effects of Using TLR-Ligands as Immunotherapeutic Agents

Activating the immune system can be a double-edged sword because although it can result in the control of a pathogen, adverse events can sometimes occur. Certain infections have been shown to trigger autoimmune diseases (102–104) and there is a possibility that the use of TLR-ligands derived from pathogenic agents may accelerate the development of autoimmunity in susceptible individuals. For example, CpG-ODNs activating TLR-9 have been implicated in triggering rheumatoid arthritis, systemic lupus erythematosus, and diabetes (105–108). Additionally, stimulation of TLR-3 has been associated with lupus nephritis (109).

TLR-7 ligands were developed with a view to treat hepatitis C virus (HCV) infections, however, a single intra-peritoneal administration of R-848 (TLR-7/8 ligand) in mice lead to a rapid depletion of leukocytes in the blood (110). Depletion of leukocytes from blood lasted for 24 h and was attributed to the retention of these cells in peripheral organs (110). Another TLR-7 agonist, PF-4878691 also intended for the treatment of HCV, caused adverse effects, which included hypotension, lymphopenia, and flu-like symptoms in patients (111).

## Conclusion

Here, we have reviewed studies in which TLR agonists have been used to stimulate the innate immune system as a way to combat infectious diseases. Immunomodulating agents are inherently non-specific in their activation of the innate immune system and the caveat to this form of treatment is the need to balance protective inflammatory anti-microbial activity against pathogen-induced damage to the host. Furthermore, the delivery of these agents should not increase host susceptibility to re-infection or secondary infection. Therefore, understanding how or if the deployment of these immunomodulatory agents affect the development of acquired immune memory responses will be important in determining the development and evolution of these agents for clinical use. Overall, they should provide immediate protection and not interfere with, the generation of long-term humoral and cell-mediated immune responses.

Determining the appropriate route of delivery is also important in the development of these immunostimulatory agents. Some of the agents that we have discussed offer immediate protection which is independent of the route of inoculation while others are only effective when administered to the target organ such as the lung. One advantage of targeting organs like the lung, is the possibility that agents could be effectively self-administered, i.e., by intra-nasal administration, removing the need for medical expertise. This feature would be highly valuable in the event of an influenza pandemic, for example, where large-scale administration is necessary and medical facilities are limited.

Host-directed immunotherapies are a promising new approach in combating infectious diseases. With the development of anti-microbial resistance to commonly used antibiotics and the need to update vaccines to accommodate pathogen evolution, agents stimulating the immune system have the potential to be developed as an important new member of our anti-microbial armamentarium in preventing and maintaining public health.

## Conflict of Interest Statement

The authors declare that the research was conducted in the absence of any commercial or financial relationships that could be construed as a potential conflict of interest.
